# Significant association between glycemic status and increased estimated postglomerular resistance in nondiabetic subjects – study of inulin and para-aminohippuric acid clearance in humans

**DOI:** 10.14814/phy2.12321

**Published:** 2015-03-05

**Authors:** Mari Yasumoto, Akihiro Tsuda, Eiji Ishimura, Hideki Uedono, Yoshiteru Ohno, Mitsuru Ichii, Akinobu Ochi, Shinya Nakatani, Katsuhito Mori, Junji Uchida, Masanori Emoto, Tatsuya Nakatani, Masaaki Inaba

**Affiliations:** 1 Department of Nephrology, Department of Metabolism, Endocrinology and Molecular Medicine, Osaka City University Graduate School of MedicineOsaka, Japan; 2 Department of Urology, Osaka City University Graduate School of MedicineOsaka, Japan

**Keywords:** Clearance, glomerular hemodynamic, glycemic control, inulin, para-aminohippuric acid, postglomerular resistance

## Abstract

We investigated whether glomerular hemodynamic parameters in nondiabetic subjects, including healthy subjects, are associated with glycemic status indices, by simultaneous measurement of inulin (*C*_in_) and para-aminohippuric acid (*C*_PHA_) clearance. Twenty-six subjects (age 49.5 ± 13.3 years; 13 men and 13 women; 14 healthy subjects and 12 subjects with mild proteinuria) were enrolled. *C*_in_ and *C*_PAH_ were measured simultaneously. All 26 subjects were nondiabetics. Estimated preglomerular resistance, estimated postglomerular resistance, and estimated glomerular hydrostatic pressure (*P*_glo_) were calculated according to Gomez’ formula. *P*_glo_ correlated significantly and positively with hemoglobin A1c (HbA1c) in both healthy subjects (*r* = 0.532, *P* = 0.0498) and subjects with mild proteinuria (*r* = 0.681, *P* = 0.015). While there was no significant correlation between estimated preglomerular resistance and HbA1c, estimated postglomerular resistance correlated significantly and positively with HbA1c both in healthy subjects (*r* = 0.643, *P* = 0.013) and subjects with mild proteinuria (*r* = 0.589, *P* = 0.044). Glomerular filtration fraction, estimated *P*_glo_ and estimated postglomerular resistance in total subjects were associated significantly with HbA1c after adjustment for age, gender, and body mass index. These results demonstrate that, even in nondiabetic subjects, glycemic status is associated with estimated postglomerular resistance, but not estimated preglomerular resistance. It is suggested that increased estimated postglomerular resistance associated with higher HbA1c levels, even within the normal range, causes increased estimated *P*_glo_, leading to increased FF. Thus, hemodynamic abnormalities associated with higher HbA1c levels may be related to glomerular hypertension, even in nondiabetic subjects.

## Introduction

It has been reported that the development and progression of diabetic nephropathy is associated with glomerular hypertension and glomerular hyperfiltration, which are induced by increased intrarenal renin angiotensin activation, atrial natriuretic peptide, and nitric oxide (Arima and Ito 2003; Lewko et al. 2004; Peti-Peterdi et al. 2008). Glomerular hypertension and hyperfiltration has been demonstrated in both type 1 and type 2 diabetes (Kanwar et al. 2008; Peti-Peterdi et al. 2008; Helal et al. 2012). However, precise glomerular hemodynamic abnormalities have not been demonstrated, particularly in humans. Glomerular hemodynamics can be examined using Gomez's formula (Gomez 1951; Guidi et al. 2001), in which both inulin and para-aminohippuric acid (PAH) clearance are measured simultaneously. We recently reported a significant association between poor glycemic control and increased efferent arteriolar resistance in diabetic patients (Tsuda et al. 2014b). However, to date, no data exist regarding the relationship between glycemic control and glomerular hemodynamics in nondiabetic subjects.

In this study, we examined glomerular hemodynamics by simultaneously measuring the clearance of inulin (*C*_in_) and para-aminohippuric acid (PAH) (*C*_PAH_). We aimed to determine whether glycemic control indices affected glomerular hemodynamics in nondiabetic subjects.

## Materials and Methods

### Subjects

The study protocol was approved by the Ethics Committee of Osaka City University Graduate School of Medicine (#1444). The subjects who had mild proteinuria (urine protein ≤ 1 + by the dip-stick test, no hematuria) or intended to provide a kidney for transplantation were admitted to Osaka City University Hospital between January 2013 and May 2014. None of the 26 subjects met the diagnostic criteria of diabetes or borderline diabetes. After obtaining written informed consent from each subject, we examined *C*_in_ and *C*_PAH_ from a total of 40 subjects. Since the original Gomez's formula could be applied to those with *C*_in_ of more than 60 mL/min (Gomez 1951), *C*_in_ values less than 60 mL/min (*n* = 14) were excluded from the analyses. After exclusion, 14 healthy subjects who intended to provide a kidney for transplantation and 12 subjects with mild proteinuria (urine protein ≤ 1 + by the dip-stick test, no hematuria and *C*_in_ > 60 mL/min) were enrolled (49.5 ± 13.3 years; 13 men and 13 women).

During the course of admission, including the study period, all participants ingested sodium 6 g/day and protein 60–70 g/day, according to the “Dietary recommendations for kidney disease, 2007”, published by the Japanese Society of Nephrology (Nakao et al. 2007). The clearance study was performed in the morning after overnight fasting (approximately 12 h fasting).

### Measurements of *C*_in_ and *C*_PAH_, and calculation of intrarenal hemodynamic parameters

Glomerular filtration rate (GFR), as measured by *C*_in_, and renal plasma flow (RPF), as measured by *C*_PAH_, were determined by the input clearance technique with inulin and PAH, respectively. According to the method by Horio et al. (2009) and the method reported previously by us (Tsuda et al. 2013, 2014a,b), inulin and PAH were administered by continuous intravenous infusion via the forearm antecubital vein in the morning, after fasting. *C*_in_ and *C*_PAH_ were measured simultaneously according to the method of Horio et al. (2009), that is, a simple method of *C*_in_ and *C*_PAH_ by single urine collection, as we have reported previously (Tsuda et al. 2013, 2014a,b). In brief, the subjects received 500 mL of water orally 15 min before the infusion. After administration of a priming bolus of inulin and PAH that was adjusted to 1 and 0.5%, respectively, with saline, the rate of infusion was 300 mL/h for the first 30 min and 100 mL/h thereafter. The subjects completely emptied their bladders 45 min after the start of the test. At the beginning of the clearance period, a blood sample was collected for serum inulin and PAH. To maintain hydration, 180 mL of water was given. At the end of the clearance period, a blood sample was drawn for serum inulin and PAH, together with the urine collection for the measurement of urinary inulin and PAH concentration. A urine collection period of 90 min was set, in order to increase the accuracy of the clearance study. *C*_in_ and *C*_PAH_ were calculated by the UV/P method (U: concentration in urine, V: urine volume [mL/min], P: concentration in plasma) using the mean value of the serum inulin concentrations at the beginning and end of the clearance period. Inulin concentrations were measured enzymatically (Horio et al. 2009; Kimata et al. 2009). PAH concentrations were measured spectrophotometrically, by means of the N-1 naphthylethylenediamine and the anthrone method using a Corning 258 spectrophotometer (Fliser et al. 2000). The clearance values were not corrected for body surface area, in part because of the report by Turner and Reilly that suggested that indexing renal hemodynamic variables by body surface may lead to inappropriate inferences and obscure gender-related differences (Turner and Reilly 1995).

As the direct measurement of glomerular hemodynamics parameters in humans is not feasible, the formulae introduced by Gomez (1951) allow indirect assessment of glomerular hemodynamics, which has been recently discussed in detail by Guidi et al. (2001). These formulae were designed for the quantitative estimation of filtration pressure across the glomerular capillaries, estimated glomerular hydrostatic pressure (*P*_glo_), and estimated preglomerular resistance and estimated postglomerular resistance using the measured blood pressure, GFR, RPF, hematocrit, and plasma protein concentrations under the assumption that (Arima and Ito 2003) intrarenal vascular resistance is divided into three compartments: afferent, efferent, and venular; (Brenner et al. 2001) hydrostatic pressures in the venules, interstitium, renal tubules, and Bowmans space are in equilibrium at approximately 10 mmHg; (Cherney et al. 2010) the filtration coefficient is estimated as 0.0406 mL/sec per mmHg per kidney and (Deferrari et al. 2002) filtration disequilibrium is postulated along the glomerular capillaries.

The Gomez formulae were calculated from the original report as follows:

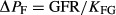


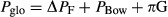


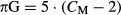


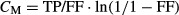


In the above, Δ*P*_F_ was the filtration pressure across the glomerular capillary. *K*_FG_ (the gross filtration coefficient) was estimated as 0.0406 mL/sec/mm Hg per kidney. *P*_Bow_ (the hydrostatic pressure in Bowman's space) was estimated as 10 mm Hg; *π*G (the oncotic pressure within the glomerular capillaries) can be obtained from *C*_M_ (plasma protein concentration within the glomerular capillaries), and calculated from the TP (total protein concentration) and filtration fraction (FF).

From Ohm's law:

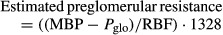


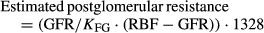


RBF can be calculated from RPF and hematocrit (Ht) using the standard formula:

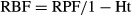


In the above, the conversion factor to dyne/sec/cm^5^ is 1328. GFR (glomerular filtration rate), RPF (renal plasma flow), and RBF (renal blood flow) are expressed in mL/sec; and the mean blood pressure (MBP) is calculated as (2 × diastolic BP + systolic BP)/3.

### Statistical methods

Results are expressed as the mean ± standard deviation (SD). Correlations between two variables were examined using Pearson's correlation coefficient. In this study, we used ordinary least squares regression. Multiple regression analysis was performed to detect predictors for filtration fraction, estimated *P*_glo_ and estimated postglomerular resistance after adjustment for other variables. All analyses were performed using StatView 5 (SAS Institute Inc., Cary, NC) for Windows. The two-sided level of statistical significance was set at *P *<* *0.05.

## Results

The baseline characteristics of the 26 subjects enrolled in this study are shown in Table1. Their mean age was 49.5 ± 13.3 years; 13 (50.0%) were male. The mean serum creatinine level was 0.76 ± 0.19 mg/dL. The measured glomerular filtration rate based on the *C*_in_ was 94.9 ± 23.8 mL/min; that is, >60 mL/min in all subjects. The fasting plasma glucose and hemoglobin A1c values were 91 ± 11 mg/dL and 5.4 ± 0.4%, respectively. There was no history of cardiovascular disease in any of the 26 subjects. Among the 14 healthy subjects who intended to provide a kidney for transplantation, none of the subjects were treated with antihypertensives. Among the 12 subjects with mild proteinuria, eight subjects were treated with antihypertensives; of these, 6, 2, and 2 were treated with angiotensin receptor blockers (ARB), angiotensin-converting enzyme inhibitors (ACEI), and calcium channel blockers (CCB), respectively.

**Table 1:** Clinical characteristics of the 26 subjects

	Mean	Range
Age (years)	49.5 ± 13.3	22–70
Healthy subjects/subjects with mild proteinuria	14/12	
Gender (male/female)	13/13	
Body mass index (kg/m^2^)	24.6 ± 4.8	18.7–36.0
Mean blood pressure (mmHg)	94 ± 10	73–114
Systolic pressure (mmHg)	127 ± 16	98–160
Diastolic pressure (mmHg)	78 ± 11	60–98
Plasma glucose (mg/dL)	91 ± 11	73–128
Hemoglobin A1c (%)	5.4 ± 0.4	4.6–6.3
Inulin clearance (mL/min/1.73 m^2^)	94.9 ± 23.8	60.2–153.3
Renal plasma flow (mL/min)	496.8 ± 171.8	291–873
Renal blood flow (mL/min)	810.4 ± 278.6	473–1451
Estimated preglomerular resistance (dyne/sec/cm^5^)	3778 ± 2856	29–9473
Estimated postglomerular resistance (dyne/sec/cm^5^)	1596 ± 1195	153–4053
Filtration fraction	0.20 ± 0.06	0.08–0.30
Glomerular hydrostatic pressure (mmHg)	52.0 ± 8.1	38.8–68.1
ARB/ACEI/CCB (only in subjects in mild proteinuria)	6/2/2	

ARB: angiotensin receptor blocker, ACEI; angiotensin-converting enzyme, CCB; calcium channel blocker.

**Figure 1:**
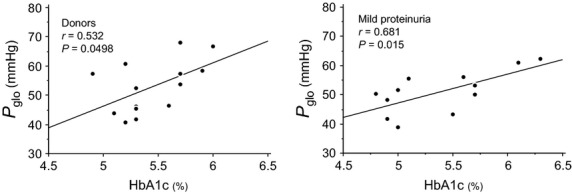
Relationship between hemoglobin A1c and filtration fraction and the glomerular hydrostatic pressure (*P*_glo_) in 14 healthy subjects (donor) and in 12 subjects with mild proteinuria. There were significant and positive correlations between hemoglobin A1c and the filtration fraction and the glomerular hydrostatic pressure in both subjects.

The relationships between the estimated *P*_glo_ and the study parameters were examined. There were no significant correlations between estimated *P*_glo_ and age, blood pressure, or albumin neither in 14 healthy subjects nor 12 subjects with mild proteinuria. Estimated *P*_glo_ was not significantly different between males and females. Estimated *P*_glo_ correlated significantly and positively with hemoglobin A1c in 14 healthy subjects (*r* = 0.532, *P* = 0.0498, Fig.1), and in 12 subjects with mild proteinuria (*r* = 0.681, *P* = 0.016). There was no significant correlation between estimated *P*_glo_ and fasting plasma glucose neither in 14 healthy subjects nor 12 subjects with mild proteinuria. In total subjects, there was a significant and positive correlation between estimated *P*_glo_ and body mass index (*r* = 0.411, *P* = 0.046). In total subjects, estimated *P*_glo_ was associated significantly with hemoglobin A1c after adjustment for age, gender, and body mass index (Table2).

**Table 2:** Factors associated with estimated postglomerular resistance, estimated *P*_glo_, and filtration fraction (FF), in all subjects

	Postglomerular resistance		Estimated *P*_glo_		FF	
	*β*	*P*	*β*	*P*	*β*	*P*
Age	−0.007	0.967	−0.048	0.783	0.281	0.143
Gender (male = 0, female = 1)	0.274	0.179	0.176	0.363	−0.297	0.160
Body mass index (kg/m^2^)	0.196	0.358	0.373	0.076	−0.151	0.491
Hemoglobin A1c (%)	0.475	0.025	0.438	0.031	0.476	0.029
*R*^2^/*P*	0.400/0.024		0.445/0.012		0.359/0.044	

**Figure 2:**
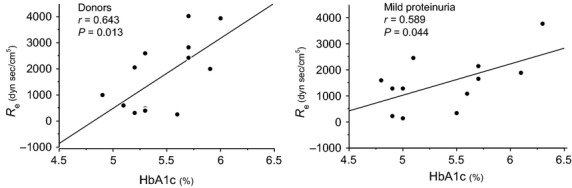
Relationship between hemoglobin A1c and estimated postglomerular resistance (*R*_e_) in 14 healthy subjects (donors) and in subjects with mild proteinuria. There was a significant positive correlation between hemoglobin A1c and the *R*_e_ both in 14 healthy subjects (donors) and in 12 subjects with mild proteinuria.

The relationships between the estimated pre- and postglomerular resistance and the study parameters were examined. There were no significant correlations between estimated preglomerular resistance and the glycemic control indices; that is, fasting plasma glucose or hemoglobin A1c neither in 14 healthy subjects nor in 12 subjects with mild proteinuria, However, estimated postglomerular resistance correlated significantly and positively with hemoglobin A1c both in 14 healthy subjects (*r* = 0.643, *P* = 0.013) and in subjects with mild proteinuria (*r* = 0.589, *P* = 0.044) (Fig.2). Estimated postglomerular resistance was associated significantly with hemoglobin A1c after adjustment for age, gender, and body mass index (Table2).

The relationships between the filtration fraction and the study parameters were examined. While the filtration fraction correlated significantly and positively with hemoglobin A1c in total subjects (*r* = 0.427, *P* = 0.030), there were no statistical significant correlation neither in 14 healthy subjects (*r* = 0.317, *P* = 0.269)) nor in 12 subjects with mild proteinuria (*r* = 0.498, *P* = 0.099). There was a significant and positive correlation between filtration fraction and age in total subjects (*r* = 0.401, *P* = 0.042). The filtration fraction was not significantly different between males and females. There were also no significant correlations between the filtration fraction and albumin, or body mass index. In total subjects, the filtration fraction was associated significantly with hemoglobin A1c after adjustment for age, gender, and body mass index (Table2).

We examined the differences in the estimated pre- and postglomerular resistance among the subjects with and without proteinuria. Neither estimated pre- nor postglomerular resistance showed any significant differences between those with and without proteinuria.

We examined the differences in estimated pre- and postglomerular resistance among subjects with and without antihypertensive therapy in 12 subjects with proteinuria, including ARB, ACEI, and CCB. Neither estimated pre- nor postglomerular resistance showed any significant differences between subjects with and without antihypertensives, that is, ARB, ACEI, or CCB. There were no significant correlations between urinary protein and estimated pre- and postglomerular resistance, estimated *P*_glo_, or filtration fraction.

## Discussion

We previously demonstrated that poor glycemic control in diabetic patients is associated with increased estimated postglomerular resistance, but not estimated preglomerular resistance. Furthermore, we suggested that the increase in the estimated postglomerular resistance causes increased estimated *P*_glo_, leading to increased FF (Tsuda et al. 2014b). In this study, we examined *C*_in_ and *C*_PAH_, and calculated intrarenal hemodynamic parameters, as in our previous study of diabetic patients (Tsuda et al. 2014b), using Gomez's formula in 14 healthy subjects and 12 subjects with mild proteinuria. GFR, as measured by *C*_in_, was greater than 60 mL/min in all subjects. We found that estimated *P*_glo_ correlated significantly and positively with the glycemic control indicator, hemoglobin A1c both in 14 healthy subjects and in 12 subjects with mild proteinuria. In nondiabetic subjects, while the estimated postglomerular resistance correlated significantly with hemoglobin A1c both in 14 healthy subjects and in 12 subjects with mild proteinuria, the estimated preglomerular resistance did not. These results indicate that glycemic control is associated with increased estimated postglomerular resistance, and suggest that the increased estimated postglomerular resistance may, in turn, be associated with increased estimated *P*_glo_, even in nondiabetic subjects. Thus, a higher glycemic state, even within the normal range, likely increases the burden on the glomeruli, possibly affecting the deterioration of the glomeruli in both diabetic and nondiabetic subjects.

Increased glomerular filtration rate has been reported in diabetic nephropathy, (Arima and Ito 2003; Komers et al. 2007; Kanwar et al. 2008). Glomerular hyperfiltration is observed in the early stage of most patients with diabetes mellitus and is thought to precede the development of microalbuminuria by several years (Arima and Ito 2003; Komers et al. 2007; Kanwar et al. 2008). In animals, the increase in glomerular filtration rate in diabetes is caused by imbalances of afferent and efferent arteriolar tone with a disproportionate decrease in afferent arteriolar resistance and a relatively higher efferent arteriolar tone, leading to an increase in glomerular capillary pressure (Hostetter et al. 1982; Komers and Anderson 2000; Komers et al. 2007). However, imbalances between afferent and efferent arteriolar resistance have not been demonstrated clinically in humans. Therefore, we recently demonstrated that poor glycemic control correlates with glomerular hemodynamic abnormalities in humans (Tsuda et al. 2014b). In our previous study of diabetic patients, we showed that poor glycemic control increased the filtration fraction, estimated *P*_glo_ and estimated postglomerular resistance, but not estimated preglomerular resistance (Tsuda et al. 2014b). All of these reports, however, have examined diabetic patients or diabetic animals. To date, there has been no report in which renal hemodynamic abnormalities were evaluated by *C*_in_ and *C*_PAH_ with respect to the relationship between glomerular hemodynamic and glycemic control indices in humans without diabetes mellitus. This is the first study to demonstrate that glycemic control is associated with glomerular hemodynamic changes in nondiabetic subjects.

From the findings of this study, the mechanism underlying increased *P*_glo_, and estimated postglomerular resistance under the status of glycemic control remains unknown. Evidence suggests that hyperglycemia could activate the intrarenal renin angiotensin system (RAS) (Leehey et al. 2000; Nishiyama et al. 2002a,b; Singh et al. 2003; Ritz and Dikow 2006; Peti-Peterdi et al. 2008; Visavadiya et al. 2011). Angiotensin II activation increases efferent arteriolar resistance, leading to glomerular hypertension and hyperfiltration (Miller 1999; Kanwar et al. 2008; Cherney et al. 2010). Several studies have shown that the RAS inhibitors, angiotensin-converting enzyme inhibitors or angiotensin II type 1 (AT1) receptor antagonists, ameliorate renal damage in diabetic animal models (Kelly et al. 2007; Feldman et al. 2008) or slow disease progression in humans (Brenner et al. 2001; Viberti and Wheeldon 2002; Parving et al. 2008). These beneficial effects of RAS inhibitors in the prevention of diabetic renal disease suggest that angiotensin II, acting through the AT1 receptor, is a major mediator of progressive renal injury (Brenner et al. 2001; Deferrari et al. 2002; Viberti and Wheeldon 2002; Kowey et al. 2005). These studies also support the activated state of intrarenal RAS in the early stage of diabetic nephropathy. From the results of this study, we consider that intrarenal RAS activation is induced even with mild glycemic abnormalities.

There are some limitations to this study. First, the study was performed in a small number of Japanese subjects. A large-scale study is needed to confirm that the relationship between glycemic control indices and glomerular hemodynamics is maintained in nondiabetic subjects. Secondly, in multiple regression analyses in this study, both healthy subjects and subjects with mild proteinuria were included in the models. This is because the number of subjects examined in this study was small. However, there were significant and positive correlations between estimated postglomerular resistance and HbA1c both in healthy subjects (*r* = 0.643, *P* = 0.013) and subjects with mild proteinuria (*r* = 0.589, *P* = 0.044). Furthermore, there were significant and positive correlations between estimated *P*_glo_ and HbA1c in both healthy subjects (*r* = 0.532, *P* = 0.0498) and subjects with mild proteinuria (*r* = 0.681, *P* = 0.015). Accordingly, we combined 14 healthy subjects and 12 subjects in multiple regression analyses in this study, as 12 subjects with mild proteinuria did not seem to have glomerulonephritis or distinct glomerular diseases. Thirdly, our calculation of pre- and postglomerular resistance and glomerular capillary pressure in humans using measurements of GFR and RBF cannot be independently verified, although it was not possible to directly measure intrarenal hemodynamic parameters in humans, as compared with animal experiments. However, according to the human studies by others (Guidi et al. 2001; Skrtic et al. 2014) and us (Tsuda et al. 2013, 2014b), these intrarenal hemodynamic parameters were calculated and estimated using Gomez's formulae in this study.

In conclusion, in this study, by measuring *C*_in_ and *C*_PAH_ in human nondiabetic subjects, we showed that the status of increased hemoglobin A1c led to increased *P*_glo_, and postglomerular resistance in nondiabetic subjects. These hemodynamic burdens may cause injury to the glomeruli, even in nondiabetic subjects.

## Conflict of Interest

None declared.
